# Visual information is required to reduce the global effect

**DOI:** 10.3758/s13414-020-01992-6

**Published:** 2020-02-12

**Authors:** Kiki Arkesteijn, Mieke Donk, Jeroen B. J. Smeets, Artem V. Belopolsky

**Affiliations:** 1 Department of Experimental and Applied Psychology, Vrije Universiteit Amsterdam, Van der Boechorststraat 7, 1081 BT Amsterdam, Netherlands; 2 Department of Human Movement Sciences, Vrije Universiteit Amsterdam, Amsterdam, Netherlands

**Keywords:** Eye movement, Selection, Averaging, Accuracy, Motor plan

## Abstract

When a distractor appears in close proximity to a saccade target, the saccadic end point is biased towards the distractor. This so-called global effect reduces with the latency of the saccade if the saccade is visually guided. We recently reported that the global effect does not reduce with the latency of a double-step memory-guided saccade. The aim of this study was to investigate why the global effect in memory-guided saccades does not show the typically observed reduction with saccadic latency. One possibility is that reduction of the global effect requires continuous access to visual information about target and distractor locations, which is lacking in the case of a memory-guided saccade. Alternatively, participants may be inclined to routinely preprogram a memory-guided saccade at the moment the visual information disappears, with the result that a memory-guided saccade is typically programmed on the basis of an earlier representation than necessary. To distinguish between these alternatives, two potential targets were presented, and participants were asked to make a saccade to one of them after a delay. In one condition, the target identity was precued, allowing preprogramming of the saccade, while in another condition, it was revealed by a retro cue after the delay. The global effect remained present in both conditions. Increasing visual exposure of target and distractor led to a reduction of the global effect, irrespective of whether participants could preprogram a saccade or not. The results suggest that continuous access to visual information is required in order to eliminate the global effect.

Objects around us are constantly competing for visual selection. People make two to three ballistic eye movements, or saccades, per second to select relevant information from their surroundings. To accurately perform a saccade, the eye movement system has to calculate a motor plan on the basis of visual information. Even though saccades typically undershoot a target by about 10% (Kapoula, 1985), their direction is quite accurate for isolated targets. However, when a competing distractor appears near a saccade target, the saccadic end point is often biased towards the distractor. Coren and Hoenig (1972) were the first to accurately describe this phenomenon and to point out that when observers have to make an eye movement to a target stimulus surrounded by irrelevant stimuli, the eyes tend to land on the center of gravity of all stimuli. This tendency to saccade towards the intermediate position between a target and a distractor is currently referred to as the global effect (Findlay, 1982; see also, e.g., Arkesteijn, Smeets, Donk, & Belopolsky, 2018; Coëffé & O’Regan, 1987; Van der Stigchel & Nijboer, 2011).

The global effect has been explained in terms of a weighted average of the peaks of activations on the saccade map, corresponding to the locations of the target and the distractor (Godijn & Theeuwes, 2002; Meeter, Van Der Stigchel, & Theeuwes, 2010; Van der Stigchel & Nijboer, 2011). According to this account, the saccade’s end point is located at the weighted average of these activations. The saccade map is presumed to be located at the intermediate layers of the superior colliculus; these layers receive input from various cortical areas such as the dorsolateral prefrontal cortex and the frontal eye fields (Goldman & Nauta, 1976; Johnston & Everling, 2008). The integrated inputs are sent to the brain stem and cerebellum, where the saccade is programmed (Meeter et al., 2010; Munoz, Dorris, Pare, & Everling, 2000; Trappenberg, Dorris, Munoz, & Klein, 2001). The weights assigned to the target and distractor locations depend on the relative salience of the two objects: Saccades are often biased towards the more salient element (Findlay, Brogan, & Wenban-Smith, 1993).

The strength of the activation in the saccade map is not only assumed to be affected by stimulus-driven factors but can also be modulated by top-down information (Meeter et al., 2010). If the activity peak associated with the target becomes stronger than that of the distractor as a result of such a top-down modulation, the saccade will be more accurately directed towards the target. One of the input areas of the superior colliculus (the dorsolateral prefrontal cortex) is susceptible to task demands, as it responds to targets, but not distractors (Everling, Tinsley, Gaffan, & Duncan, 2006). Therefore, this area might provide the top-down information.

As top-down modulations typically affect saccades with longer latencies (Van Zoest, Donk, & Theeuwes, 2004), only short-latency saccades will be primarily directed towards the weighted average, whereas the effect of the distractor will be reduced for long-latency saccades (Coëffé & O’Regan, 1987; Findlay, 1982; Heeman, Theeuwes, & Van der Stigchel, 2014; for an overview, see Van der Stigchel & Nijboer, 2011). For instance, Heeman et al. (2014) presented participants with two targets and instructed them to saccade towards a prespecified target. They found that the global effect decreased with longer latencies, and was even abolished with saccade latencies longer than 330 ms. However, when no target was prespecified (i.e. the participant could choose to saccade to either target), the global effect remained stable.

Even though the global effect has been consistently found to disappear when saccade latencies were longer than approximately 300 ms (Heeman et al., 2014; Ottes, Van Gisbergen, & Eggermont, 1985), a recent study showed that it does not reduce when participants had to keep the target in memory across a saccade (Arkesteijn et al., 2018). In Arkesteijn et al. (2018) participants had to execute two consecutive saccades while the second saccade target had to be kept in memory. The target–distractor competition was updated across the first saccade, and the size of this global effect did not change following longer saccade latencies. Thus, even though the global effect declines with saccade latency in visually guided saccades (Heeman et al., 2014; Ottes et al., 1985), it was not the case when the target was memorized (Arkesteijn et al., 2018).

One possibility is that target–distractor competition can be resolved even in the absence of visual information, but observers typically refrain from doing so because they routinely preprogram a saccade at the moment the visual information disappears and well before it is executed (Abrams & Jonides, 1988; Findlay & Walker, 1999). Alternatively, it is possible that target–distractor competition cannot be resolved in the absence of visual information, because the required weight changes in the saccade map can only be realized in the presence of visual information. This would imply that even if these weight changes might be due to top-down processing, the presence of a visual signal is required.

The aim of the present study is to distinguish between these two possibilities by comparing memory-guided saccades in two experimental conditions. In both conditions, participants were briefly presented with two potential targets and two distractors and were asked to make a saccade to one of the targets after the stimuli disappeared. In the “known” condition, the color of saccade target was precued before the stimuli appeared. Therefore, the precue allowed for the memory-guided saccade to be preprogrammed based on the available visual information. Conversely, in the “unknown” condition, the color of the target was retro cued after the stimuli disappeared. Here, a memory-guided saccade had to be programmed from memory, only after the retro cued revealed the identity of the saccade target. If the persistence of the global effect over time for memory-guided saccades is due to preprogramming a saccade, the global effect should be resolved in the “unknown condition,” in which such a possibility was precluded. If the persistence of the global effect over time for the memory-guided saccades is due to the inability to reduce the bias from memory, the global effect should still be present in both conditions. This would suggest that continuous access to visual information is pivotal for resolving the global effect.

## Experiment 1

### Method

#### Participants

Twenty-four participants of the Vrije Universiteit Amsterdam were recruited to take part in the experiment. Twenty-one participants completed all blocks of trials. The data of three participants were later excluded because of excessive data loss (>50%) as a result of eye-tracking errors. Eighteen participants (age range: 18–26 years, mean = 21, 15 women) were included in the data analysis. All had normal or corrected-to-normal vision and were naïve to the purpose of the study. Informed consent was obtained from all participants, and the experiment was approved by the Ethical Committee of the faculty of Behavioural and Movement Sciences of the Vrije Universiteit Amsterdam.

#### Apparatus

The experiment was conducted in a dimly lit room. A 21-in. LCD monitor (Samsung 2233RZ) with a 1,680 × 1,050-pixel resolution and a 120 Hz refresh rate displayed the stimuli. An EyeLink 1000 (SR research) recorded gaze with a temporal resolution of 1 ms and a spatial resolution of 0.01°. Experimental software was written to control the stimulus presentation, response collection, and eye tracking in OpenSesame, Version 2.9 (Mathôt, Schreij, & Theeuwes, 2012), using a PsychoPy back end (Peirce, 2007) and PyGaze (Dalmaijer, Mathôt, & Van der Stigchel, 2014). An automatic algorithm detected saccades using minimum velocity and acceleration criteria of 35°/s and 9,500°/s^2^, respectively.

#### Stimuli and procedure

**Fig. 1:**
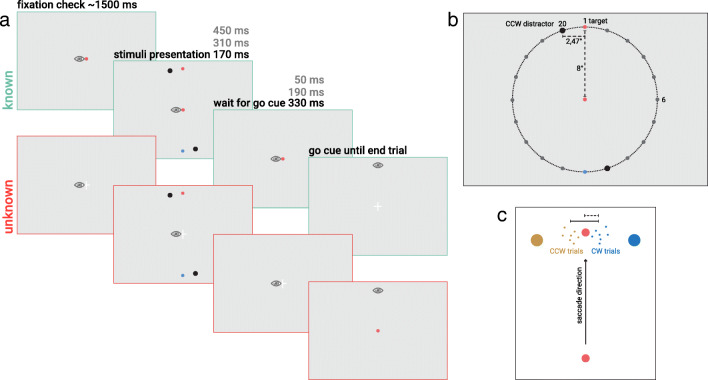
**a** Sequence of events of a trial in both conditions. Participants had to saccade to the target (color indicated by the fixation dot in the known condition) after a delay of 330 ms (50, 190, or 330 ms in Experiment 2) after the disappearance of the peripheral stimuli. In the “unknown” condition, the initial fixation was the white cross, and 330 ms (50, 190, or 330 ms in Experiment 2) after the disappearance of the peripheral stimuli, the white cross turned into a blue or red dot, revealing the color of the saccade target and serving as the “go” cue. **b** Spatial layout of the stimuli, with 20 possible positions for the target. The irrelevant target was always positioned at the opposite side of the imaginary circle. The distractor was presented at the next neighboring position—at Position 20 for the CCW condition and at Position 2 for the CW condition. **c** Example of calculation of the landing bias (dotted line) as half of the distance between the averaged landing positions of CW and CCW trials (solid line). (Color figure online)

Participants sat with their head positioned on a chin and forehead rest at a distance of 70 cm from the display. Each session started with a nine-points calibration procedure, which we accepted when the average error was smaller than 1°. We presented the stimuli on a gray (29 cd/m^2^) background. The saccade target was either a red (15 cd/m^2^) or blue (15 cd/m^2^) dot (diameter 0.4°) and could appear at one of 20 equidistant positions on an imaginary circle (8° radius; see Fig. 1b). The colored dots were always positioned exactly opposite to each other. One black distractor (diameter 0.8°) accompanied each colored dot at one of its neighboring positions, resulting in four peripheral dots.

Participants were instructed to fixate, to remember the (potential) target location(s), and to make a saccade to the remembered location of the target as fast as possible upon the appearance of the “go: cue (see Fig. 1). In the “known” condition, the trial began with the presentation of a red or blue fixation dot (diameter: 0.4°) indicating the color of the saccade target. In the “unknown” condition, the trial began with the presentation of a white fixation cross (size: 0.4°). If fixation was not detected in a 3° range within 5 seconds, a new calibration procedure was started before the trial onset. After a variable delay (drawn from a normal distribution, μ = 1,500 ms, σ = 300 ms) after fixation was detected, the four dots (one red, one blue, and two black) appeared for 170 ms. After a delay of 330 ms after the offset of the peripheral dots, a “go” cue appeared, indicating that observers had to make a saccade towards the target. Thus, the “go” cue appeared 500 ms after stimulus onset, a latency at which the global effect was reduced to zero in experiments in which the target remained visible throughout the trial (Heeman et al., 2014; Ottes et al., 1985). In the “known” condition, the “go” cue consisted of a change of the central colored fixation dot into a white cross. In the “unknown” condition, the “go” cue consisted of a change of the central white cross into a red or blue dot, revealing the color of the saccade target.

Participants heard a brief tone (277 Hz, 100 ms) if after the saccade their gaze was not within 4° of the target. If the saccade was executed before cue onset, participants received visual feedback (telling them that their eye movement was executed too soon) in combination with a brief tone. Each participant participated in two sessions that took place on different days; one for the “known” and the other for the “unknown” condition. The order of the tested conditions was counterbalanced over participants. Each session consisted of six blocks of 80 trials (20 target locations, two distractor locations, two repetitions) and lasted one hour, resulting in a total of 960 trials per participant.

#### Data analysis

To extract all relevant details and events, eye-tracking data were analyzed off-line using custom code written in Python (Anaconda, 2019). The first saccade was a defined as a saccade (as detected by the EyeLink automatic algorithm) that was larger than 4° and that was executed after the onset of the stimuli. A trial was excluded when the saccade latency was shorter than 80 ms or longer 500 ms, calculated from the “go” cue onset. Furthermore, a trial was excluded when a saccade did not land within 4° distance of the target. This way, a total of 13,321 trials (77%) were included in the analysis. All the end points of the saccade were rotated as if all saccade targets were presented to the right of fixation (Position 1 in Fig. 1b). For instance, end points for trials in which the target was at Position 6 (most downward position) were rotated 90° in the counterclockwise direction.

To examine the influence of the distractor on the saccade end points, the landing bias was calculated. The landing bias was defined as the (signed) difference in saccade landing positions between the mean perpendicular distance when the distractor was presented counterclockwise, and when the distractor was presented clockwise, divided by two (see Fig. 1c). In this way, a positive value indicates a landing bias towards the distractor: A value of zero indicates saccades landing at the target without any vertical bias, and a negative value indicates a bias away from the distractor. This measure of the landing bias pools the data across clockwise and counterclockwise trials, without being perturbed by potential idiosyncratic biases that are independent of the distractor.

To assess whether there was an effect of the distractor on the landing position in a condition, it was determined whether the landing position was biased to the distractor (landing bias was greater than zero, alternative hypothesis) or if the landing position was unrelated to the distractor position (null hypothesis). This was done for both the “known” and the “unknown” conditions. A Bayesian one-sided, one-sample *t* test was performed using the default Cauchy prior (scale: 0.707) by JASP 0.9.2.0 (JASP Team, 2018), with the prediction that the landing bias would be greater than zero in both conditions. Likewise, we tested whether the landing bias differed between the “known” and “unknown” conditions using a Bayesian two-sided, paired-sample *t* test. The Bayes factor (BF) was reported, which indicates support for one hypothesis over another hypotheses, expressed in a likelihood estimation (Wagenmakers, Lodewyckx, Kuriyal, & Grasman, 2010). A BF_10_ is reported when there is evidence in favor of the alternative hypothesis compared with the null hypothesis, and a BF_01_ is reported when there is evidence in favor of the null hypothesis over the alternative hypothesis. A BF >3 indicates that there is substantial evidence, a BF >10 indicates that there is strong evidence, and a BF >100 indicates that there is decisive evidence for a hypothesis to be true (Raftery, 1995).

Because the magnitude of the global effect has been shown to depend on saccade latency (Findlay, 1982; Heeman et al., 2014; Ottes et al., 1985), and longer saccade latencies were expected in the “unknown” condition, we reconstructed the time course of the landing bias as a function of saccade latency for the “known” and “unknown” conditions using the SMART method (Van Leeuwen, Smeets, & Belopolsky, 2019). First, for each participant and condition, the landing position data was smoothed with a Gaussian kernel (σ = 20 ms) for latencies ranging from 200 to 400 ms. This was done both for the CW and CCW trials. Subsequently, the difference between the smoothed time series obtained in the CW and CCW trails were divided by 2 to acquire the landing bias as a function of saccade latency. Next, a weighted paired *t* test was performed between the smoothed time series for the “known” and “unknown” conditions to assess whether the landing bias was significantly different between the conditions at any time point (for a detailed description, see Van Leeuwen et al., 2019).

As the landing bias reduces linearly with saccade latency for visually guided saccades (Heeman et al., 2014), a linear regression line was fit to the smoothed time series for the “known” and “unknown” conditions and for every participant. To assess whether the size of the landing bias decreased with longer saccade latencies, two Bayesian one-sample *t* tests were performed on the slopes for both conditions.

### Results

**Fig. 2:**
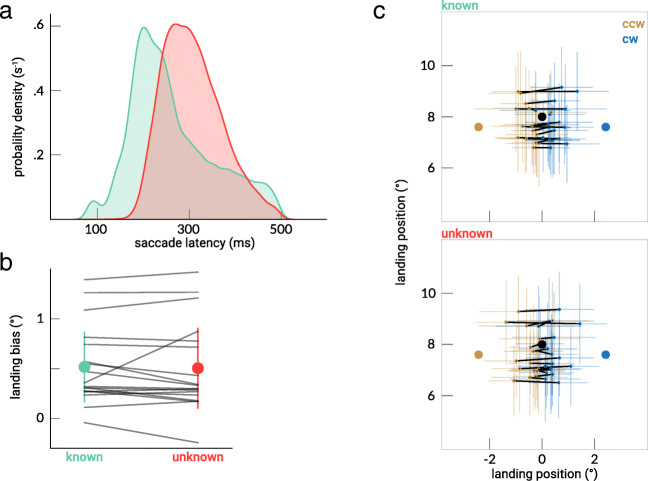
Results of Experiment 1. **a** Saccade latency distributions (smoothing kernel width = 10 ms) for the “known” and “unknown” conditions. **b** The mean landing bias in the “known” and “unknown” condition. Individual participants are indicated by separate lines; error bars indicate 95% confidence interval of the mean across participants. **c** Mean two-dimensional landing position for each participant for the CW trials (blue) and CCW trials (yellow) in the “known” condition (above) and “unknown” condition (below). Each participant’s CW and CCW trial means are connected by a black line; error bars indicate the standard deviations in *x* and *y* direction. Saccades start at 0, 0. (Color figure online)

Saccade latencies were smaller for the “known” than for the “unknown” condition (see Fig. 2a). The median latencies were 243 ms (interquartile range [IQR]: 199–321 ms) and 302 ms (IQR: 260–349 ms), respectively. The effect of the distractor on the landing position was similar in both conditions: The landing bias was 0.52 ± 0.39° in the “known” condition and 0.50 ± 0.44° in the “unknown” condition (see Fig. 2b–c). The conditions were compared using Bayesian paired *t* tests. The landing bias was greater than zero in both conditions (“known”: BF_10_ = 1377; “unknown”: BF_10_ = 314). More importantly, no evidence was found that the landing bias differed between the “known” and “unknown” conditions (BF_10_ = 0.19), and it was more likely that the landing bias was the same for both conditions (BF_01_ = 5.21).

**Fig. 3:**
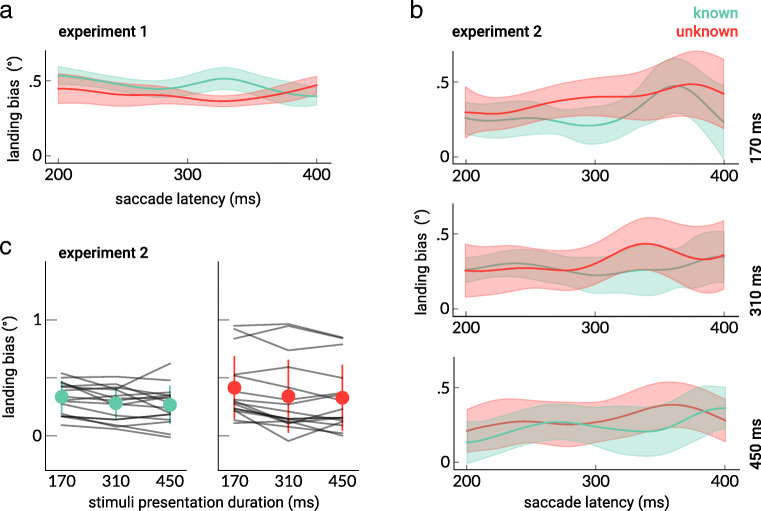
The landing bias as a function of saccade latency for Experiments 1 (**a**) and 2 (**b**), reconstructed using the SMART method. The transparent areas indicate the 95% confidence interval of the mean. **c** The mean landing bias in the “known” and “unknown” conditions. The landing bias was greater for the 170-ms presentation time compared with both 310 and 450-ms presentation times both in the “known” and “unknown” groups. The gray lines indicate individual subjects. The error bars represent the 95% between-participants confidence intervals. (Color figure online)

The time course of the landing bias is presented in Fig. 3. The SMART time-series analysis showed that the landing bias for the two conditions did not differ at any time point. Furthermore, the slopes resulting from the regression analysis did not appear to be smaller than zero in the “known” condition: −0.4 ± 3°/s (BF_10_ = 0.40) and were likely not smaller than zero in the “unknown” condition: −0.3 ± 3°/s (BF_10_ = 0.39), indicating that the landing bias most likely did not reduce over time in both conditions.

### Discussion

The results show that the global effect was present in both conditions. Because the memory-guided saccade could not be preprogrammed in the “unknown” condition, the sustained global effect in a memory-guided saccade cannot be attributed to the preprogramming of the saccade. Moreover, the size of the global effect did not change with saccadic latency. This suggests that bias towards the distractor can only be reduced in the presence of visual information. If this is the reason for the sustained global effect in memory-guided saccades, an extended presentation duration of target and distractor should lead to a reduction in the landing bias, irrespective of saccadic latency. This was tested in Experiment 2.

## Experiment 2

Previous studies using visually guided saccades showed that the global effect can be significantly reduced and even abolished when saccade latency increases (Findlay, 1982; Heeman et al., 2014; Ottes et al., 1985). For visually guided saccades, the saccade latency is roughly equivalent to the presentation time of the stimuli. If only the presence of visual information is critical for reduction of the global effect, then extending the presentation time should also reduce and possibly abolish the global effect for the memory-guided saccades. To that end, in Experiment 2, the design of Experiment 1 was extended with two additional presentation durations (i.e., in addition to 170 also 310 and 450 ms durations were used).

### Method

#### Participants

Both “known” and “unknown” conditions were tested in different groups of participants. In this way, we prevented a possible interference between the two conditions. Given the results of Experiment 1, a comparison between conditions is less relevant. Eighteen participants of the Vrije Universiteit Amsterdam took part in the “known” group. Three participants were excluded from the analysis based on the same criteria used in Experiment 1. Fifteen participants (age range: 18–25 years, mean = 21 years, 12 women) were included in the data analysis. Seventeen participants of the Vrije Universiteit Amsterdam took part in the “unknown” group. One participant was excluded from the analysis because of computer problems, and one participant was excluded from the analysis based on the criteria explained below. Fifteen participants (age range: 18–25 years, mean 21 years, 13 women) were included in the data analysis. All had normal or corrected-to-normal vision and were naïve to the purpose of the study. Informed consent was obtained from all participants, and the experiment was approved by the Ethical Committee of the faculty of Behavioral and Movement Sciences of the Vrije Universiteit Amsterdam.

#### Apparatus, stimuli, and procedure

The apparatus, stimuli, and procedure were the same as in Experiment 1, with a few exceptions. The two conditions were tested in different groups of participants. The presentation duration of the peripheral dots was varied within participants: There were three durations: 170, 310, and 450 ms. The delays between stimulus offset and the “go” cue were 330, 190, and 50 ms, respectively. Therefore, the “go” cue was always presented 500 ms after the stimulus onset. Participants in each group completed 540 trials (three durations, 10 target locations, two distractor locations, nine repetitions) in less than 1 hour of testing.

#### Data analysis

Extraction and off-line analysis of the data was the same as in Experiment 1, with the exception of the SMART analysis: To assess whether the landing bias would disappear over time, the landing bias was compared with zero for all three presentation times separately for the “known” and “unknown” groups. In the “known” group, three participants were excluded because of a loss of more than 50% of the data. A total of 6,112 (75%) trials were included in the analysis. In the “unknown” group, one participant was excluded from further analysis because of a loss of more than 50% of the data after the trials were excluded. A total of 7,200 trials (83%) were included in the analysis.

### Results

The saccade latencies are similar to the ones observed in Experiment 1. Median saccade latency was 246 ms (IQR: 198–326 ms) for the “known” group and 306 ms (IQR: 271–350 ms) for the “unknown” group.

For the “known” group, the landing bias obtained with a presentation time of 170 ms (0.33°) was larger than that with a presentation time of 310 ms (0.28°, BF_10_ = 10.5) and 450 ms (0.26°, BF_10_ = 11.4; see Fig. 3). This was also the case for the “unknown” group: The landing bias for the 170 ms presentation time (0.41°) was larger than that for the 310 ms presentation time (0.34°, BF_10_ = 8.7) and 450 ms presentation time (0.33°, BF_10_ = 258.2). For both groups, no observable differences were found between the 310 and 450-ms presentation times (BF_10_ < 3).

The SMART time-series analysis showed that the landing bias for the two groups did not differ from zero at any time point and for all presentation times (see Fig. 3). Furthermore, similar to the results in Experiment 1, no evidence was found to conclude that the slopes from the regression analysis were smaller than zero for both groups for all presentation times (all BF_10_ <3).

### Discussion

The results from Experiment 2 replicated the results from Experiment 1: The global effect did not depend on saccadic latency or on prior knowledge about the target location. In addition, the present findings show that the prolonged availability of visual information can be used to reduce the global effect: The landing bias became smaller when the presentation duration increased from 170 ms to 310 ms in both groups. Nevertheless, the global effect did not fully disappear, even when the visual information was available for as long as 450 ms, which has been shown to be sufficiently long to resolve the global effect for visually guided saccades (Heeman et al., 2014; Ottes et al., 1985). It seems that for memory-guided saccades, the influence of the distractor remains present, even when there is ample time to process visual information. This suggests that the bias towards the distractor can only be partly reduced on the basis of visual information that was present before the “go” cue.

## General discussion

The present results showed that the global effect is present and persistent in memory-guided saccades. Moreover, the size of the global effect did not reduce with increasing saccade latencies. This was the case not only in the “known” condition, in which the saccades could have been preprogrammed, but also in the “unknown” condition, in which the saccade had to be planned from memory. Importantly, the global effect was reduced when targets and distractors were presented for longer periods of time, suggesting that availability of visual information is essential for reducing target–distractor competition (Meeter et al., 2010; Van der Stigchel & Nijboer, 2011). Unlike for the visually guided saccades (Heeman et al., 2014; Ottes et al., 1985), the global effect was not completely abolished with extended presentation times for the memory-guided saccades.

These results are consistent with a previous study (Arkesteijn et al. 2018), where a global effect was found in a double-step memory-guided saccade, together with a sustained global effect as a function of saccade latency. The magnitude of the global effect for the double-step memory-guided saccades was smaller than for the single memory-guided saccades reported here (8% bias in the direction of the distractor in the previous study, compared with 20% bias reported in the present study). Therefore, the sustained time course of the global effect is not something specific to the double-step memory-guided saccades but seems to reflect a general limitation of using location information from memory.

While the persistence of the global effect in double-step saccades (Arkesteijn et al., 2018) could have been because participants had preprogrammed a saccade, this explanation was not supported by results obtained in the present study. The similarity of the time course of the global effect in the conditions when the identity of the target was known in advance or only revealed later is remarkable. In both cases the distractor influenced saccade target selection, and this influence was sustained over time. This suggests that the process of target selection from memory is very similar to the process of target selection from external world, which has been proposed in several previous studies (Belopolsky & Theeuwes, 2011; Munneke, Belopolsky, & Theeuwes, 2012).

The global effect did not fully disappear and remained present even in the presence of ample viewing and preparation time, in contrast to previous studies using visually guided saccades (Heeman et al., 2014; Ottes et al., 1985). The crucial difference seems to lie in the availability of visual information up to the moment of saccade onset for the visually guided saccades and the absence of visual information at the moment of saccade onset for the memory-guided saccades. While extended presentation of visual information improves saccade target selection, it is not enough to fully eliminate the competition from distractor. As our experiment differed in more aspects than in the availability of target information at saccade onset, it might be worthwhile to test this suggestion directly in future experiments.

A reduction of the global effect in visually guided saccades is possibly driven by top-down modulation (Heeman et al., 2014; Meeter et al., 2010). Other examples of top-down modulation that influences the global effect include, for instance, increasing the probability of the target location (He & Kowler, 1989), as well as precueing the target location ahead of time (Aitsebaomo & Bedell, 2000). The present results suggest that top-down modulation needs a continuous availability of the visual stimuli up to the moment of saccade execution to exert its effects. This suggests that the presence of visual information is required to modify the activation pattern in the saccade map.

Similar conclusions regarding memory-guided saccades have been reached in a study by de Brouwer, Brenner, Medendorp, and Smeets (2014) using a completely different paradigm. They explored the influence of Muller–Lyer illusion on accuracy of the double-step memory-guided saccades. This visual illusion biased saccade landing positions, and an increased presentation time reduced the illusion. However, prolonging the presentation times above 300 ms did not further reduce the illusion (de Brouwer et al., 2014). Also, in their studies, delaying saccade onset did not influence the accuracy of saccade (de Brouwer, Brenner, & Smeets, 2016).

To summarize, we demonstrate that the global effect cannot be fully extinguished in the memory-guided saccades even with ample viewing and preparation time. This is not due to the inability to adjust a preprogrammed saccade. While saccade target selection from the external world and from memory seem to have a similar time course, we conclude that continuous access to visual information up to saccade onset is essential for reducing and eliminating the global effect.

## Data Availability

The data can be accessed via this link: https://osf.io/cztyx
